# The Cost of Provisions in Institutions.—I

**Published:** 1904-04-09

**Authors:** 


### HOSPITAL ADMINISTRATION.

### CONSTRUCTION AND ECONOMICS.

### THE COST OF PROVISIONS IN INSTITUTIONS.—I.

By the Author of "Hospital Expenditure—The Commissariat."

INCREASING EXPENDITURE.

It is impossible to ignore at the present day the vital necessity of restricting hospital expenditure. The very existence of the voluntary hospitals is threatened by the ever increasing cost of maintaining them, which is throwing every year a greater strain on the resources of their supporters. Public opinion is strongly set in the direction of efficiency, and will not suffer any parsimony in the equipment of buildings and apparatus, nor any lagging behind in the costly business of scientific achievement. The cost of service in these institutions has long been reduced to a minimum. Much of the work performed is purely honorary; the stipends received by members of the permanent staff are often nominal, and even for the great majority of hospital workers the honour of serving the poor at the lowest living wage suffices. Retrenchment is not then indicated in this direction.

But in the purchase and distribution of the vast quantities of stores required in the hospital household there is still much need for economy, and it is in this direction that managers may most hopefully look for some reduction of expense. In the use of drugs, of dressings, and of all the expensive paraphernalia of the surgery there is room in nearly all hospitals for a closer vigilance, and in those hospitals where this department is diligently supervised a saving of £5 a bed has been secured. In domestic matters, too, in such humble necessaries as hardware, brushes, chandlery, etc., the effects of supervision are soon apparent, and nowhere is it more necessary. We do not propose, however, to touch on either the surgery or domestic charges.

It is with the responsible work of victualling these large establishments that we are now alone concerned, and it is perhaps necessary to say that reduction of expense in this quarter can only be advocated because it has been found by experience that in institutions economy and efficiency do unfailingly go hand in hand together.

In London hospitals the cost of provisions amounts to about one-fourth of the total ordinary expenditure. In provincial hospitals the proportion is even higher, the cost of provisions absorbing about one-third of the total ordinary expenditure. The cost of provisions in the hospitals of Great Britain, calculated on the basis of occupied beds, varies between £34 and £14 per annum, but this includes every variety of administration, the number of the persons boarded in addition to the patients being responsible for a large proportion of the difference.

The cost of the commissariat varies not only as between one hospital and another, but in the same hospital from one year to another.

Looking back through successive volumes of Burdett's *Hospitals and Charities*, the value of the figures collected laboriously year after year becomes apparent.

We have taken five specimen years and compared the rate of provisions per occupied bed in some hospitals with medical schools throughout the kingdom. The figures for 1902 are the latest now available. Those for 1891 are the earliest obtainable under a form which makes comparison serviceable.

TABLE I.—*Average Cost of Provision per Occupied Bed* (*London*).

| | 1891 | 1893 | 1897 | 1901 | 1902 | Remarks |
|---|---|---|---|---|---|---|
| | £ | £ | £ | £ | £ | |
| The London ... ... | 25 | 27 | 26 | 29 | 31 | |
| Guy's ... ... ... | 25 | 26 | 22 | 28 | 29 | |
| St. Bartholomew's ... | 27 | 24 | 24 | 26 | 25 | |
| St. Thomas's ... ... | 22 | 27 | 23 | 24 | 24 | |
| St. George's ... ... | 27 | 25 | 26 | 32 | 36 | |
| The Middlesex ... | 29 | 30 | 27 | 30 | 26 | |
| St. Mary's ... ... | 31 | 28 | 27 | 29 | 30 | |
| King's ... ... ... | 27 | 25 | 23 | 28 | 30 | |
| Charing Cross ... | 28 | 25 | 24 | 21 | 27 | |
| Royal Free ... ... | 24 | 26 | 25 | 26 | 28 | |
| Total average ... ... | 26·5 | 26·3 | 24·7 | 27·9 | 28·6 | |
| Total of occupied beds | 3,154 | — | — | — | 3,578 | |

It will be observed that sinking all differences between one hospital and another, the general trend is towards an increase of expenditure on the commissariat department. There are several causes which may legitimately contribute to raise the average cost per bed of provisions. The average cost per bed includes the cost, as we have said, not only of the patient but of all who are boarded in the hospital. Any increase in the staff will be instantly reflected in the cost of provisions, and during the last ten years a certain increase has taken place in the staff of most hospitals. Differences of administration may also cause a rise in the cost of provisions, and this without any corresponding cost to the hospital; as, for instance, when a laundry is established on the premises, and part of the expense of the washing bill is transferred to feeding the maids, or when porters and engineers are provided with meals in the hospital in lieu of board wages.

Any rise in prices will also inevitably show itself in the hospital accounts, and in the best administered institutions fluctuations in price are more likely to occur than in others where laxity of management has allowed the contractor an ample margin.

Taking all these points into consideration, it is not, however, altogether satisfactory to note the steady rise (with a curious dip for the year 1897) which has added an average of £2 per bed for provisions on to the expenses of hospitals in London. Two pounds per bed for the ten hospitals we have included in the return works out at nearly £7,000 a year.

For the eleven provincial hospitals below the rise in cost is £1 per bed, and the extra expenditure is about £2,400 a year. The number of Scotch and Irish hospitals which afford data for comparison is too small to make averages serviceable but it will be noticed that the upward trend is observable in nearly all.

TABLE II.—*Average Cost of Provisions per Occupied Bed (England, Scotland, Ireland).*

| | 1891 | 1893 | 1897 | 1901 | 1902 | Remarks |
|---|---|---|---|---|---|---|
| ENGLAND (Provincial) | £ | £ | £ | £ | £ | |
| Leeds General Infirmary ... | 19 | 18 | 19 | 22 | 21 | |
| Birmingham General ... | 18 | 20 | 24 | 21 | 20 | |
| Liverpool Royal Infirmary | 21 | 18 | 13 | 14 | 14 | |
| Manchester Royal Infirmary | 18 | 19 | 18 | 22 | 21 | |
| Newcastle Royal Infirmary | 21 | 19 | 19 | 22 | 23 | |
| Bristol Royal Infirmary ... | 18 | 18 | 22 | 22 | 23 | |
| Sheffield Royal Infirmary... | 22 | 24 | 25 | 29 | 29 | |
| Liverpool Royal, Southern | 17 | 18 | 17 | 17 | 17 | |
| Addenbroooke, Cambridge | 22 | 32 | 25 | 28 | 29 | |
| Radcliffe Infirmary, Oxford | 24 | 23 | 20 | 20 | 22 | |
| Birmingham Queen's ... | 20 | 19 | 18 | 19 | 18 | |
| Average ... ... ... | 29 | 20 | 20 | 21 | 21 | |
| Total of occupied beds ... | 2121 | — | — | — | 2374 | |
| SCOTCH | | | | | | |
| Edinburgh Royal Infirmary | 20 | 20 | 19 | 22 | 20 | |
| Glasgow Royal Infirmary... | 19 | 19 | 19 | 19 | 20 | |
| Glasgow Western Infirmary | 20 | 21 | 21 | 25 | 25 | |
| Aberdeen Royal Infirmary | 13 | 17 | 16 | 14 | 14 | |
| IRISH | | | | | | |
| Belfast Royal Hospital ... | 16 | 16 | 15 | 17 | 17 | |
| Meath General Hospital ... | 17 | 20 | 22 | 21 | 21 | |
| Adelaide Hospital, Dublin | 22 | 22 | 28 | 28 | 26 | |

It must be remembered, in comparing the cost of provisioning these London hospitals with that of others in various parts of the kingdom, that these are all hospitals with medical schools attached, and of sufficient size to require the services of a large staff of resident medical officers. They show, therefore, the average cost of food in hospitals where all the elements of a hospital household, patients, servants, nurses, doctors, are to be found. University College Hospital, where the nurses have only recently been boarded in the hospital, and Westminster Hospital, where only a proportion of the nurses are boarded, are omitted from the list. In calculating expenditure on the basis of occupied beds it may be necessary to point out that the larger the number of beds occupied in a given year, the smaller will be the cost, because the board of all the extra persons included in the household is divided among an increased number of patients. It is not true that large hospitals can be victualled at less cost than small ones. But if a new ward be opened, or a larger proportion of beds be used in a year, it ought to result in a decrease of cost per occupied bed by increasing the divisor out of proportion to the increase in the dividend.

Now it is to be observed in Table I. giving the cost for provisions per occupied bed in various years, that the number of occupied beds in these ten hospitals has increased from 3,154 in 1891 to 3,578 in 1902; yet the result is an increase of £2 instead of a decrease in the cost of provisions per occupied bed.

It will be generally considered that £7,000 is a large extra sum to spend on provisions in ten hospitals without any striking changes to account for it. Reckoning, however, that on an average there are about half as many other inmates as there are patients, the sum of £2 per bed per annum does not amount to as much as a penny a day for each person, and we are brought face to face with the tremendous importance of little sums in dealing with the hospital commissariat.

